# Venous Small Bowel Infarction: Intraoperative Laser Doppler Flowmetry Discriminates Critical Blood Supply and Spares Bowel Length

**DOI:** 10.1155/2012/195926

**Published:** 2012-10-10

**Authors:** S. A. Käser, P. M. Glauser, C. A. Maurer

**Affiliations:** Department of General, Visceral, Vascular and Thoracic Surgery, Hospital of Liestal, University of Basel, 4410 Liestal, Switzerland

## Abstract

*Introduction*. In mesenteric infarction due to arterial occlusion, laser Doppler flowmetry and spectrometry are known reliable noninvasive methods for measuring microvascular blood flow and oxygen utilisation. *Case Presentation*. As an innovation we used these methods in a patient with acute extensive mesenteric infarction due to venous occlusion, occurring after radical right hemicolectomy. Aiming to avoid short bowel syndrome, we spared additional 110 cm of small bowel, instead of leaving only 80 centimetres of clinically viable small bowel in situ. The pathological examination showed only 5 mm of vital mucosa to be left distal to the dissection margin. No further interventions were necessary. *Conclusion*. Laser doppler flowmetry and spectrometry are potentially powerful methods to assist the surgeon's decision-making in critical venous mesenteric perfusion, thus having an important impact on clinical outcome.

## 1. Introduction

Laser Doppler flowmetry (LDF) [1] and spectrometry [2] are reliable noninvasive methods for measuring viability of human tissue. LDF is a known reliable predictor of ischemic injury in acute mesenteric infarction due to arterial occlusion [3]. Mesenteric venous thrombosis is rare and most often can be treated with anticoagulants only [4]. However, if surgical treatment is required [5], the intraoperative assessment of bowel viability is hazardous, even if doppler sonography or fluorescein is used [6]. 

As an innovation we used LDF and spectrometry to assess bowel viability in a case of acute postoperative mesenteric infarction due to mesenteric venous thrombosis.

## 2. Case Presentation

**Figure 1:**
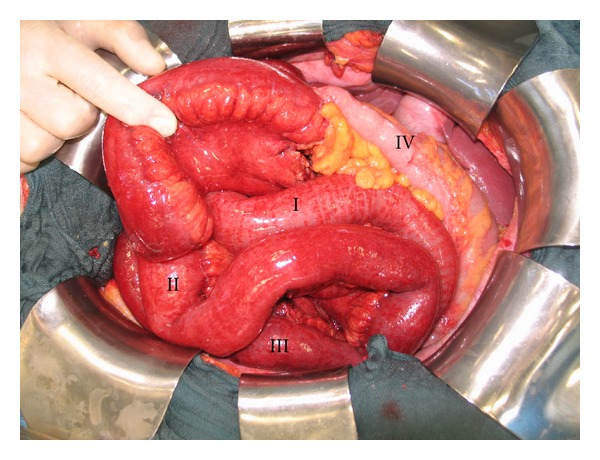
Operative situs after second-look laparotomy. The proximal segment of the jejunum (80 cm) is slightly congested but appears to be vital (I); the congested distal segment of the jejunum and the ileum have a highly questionable viability (II and III). The transverse colon (IV) has a normal appearance. The resection of the whole bowel with questionable viability would probably lead to short bowel syndrome.

Revision laparotomy due to septic condition one day after radical right hemicolectomy showed an extensive infarction of the small bowel in a fifty-one year old woman as seen in Figure 1. She had mesenteric venous thrombosis involving the ileum and the jejunum probably due to compromised blood flow in the superior mesenteric vein. The proximal part of the jejunum of about 80 cm (segment I), was slightly congested but appeared to be vital. The other part of the smaller intestine up to the ileocolic anastomosis (segments II and III) was congested and viability was highly questionable. The colon (segment IV) looked normal. Aiming to avoid short bowel syndrome, we decided to use LDF and spectrometry to save as much bowel as possible (O2C device, LF-2 probe, LEA Medizintechnik GmbH, Germany).

In this unknown situation we used general threshold values recommended by the manufacturer (microvascular haemoglobin concentration <90 units, microvascular flow >10 units, microvascular haemoglobin oxygenation >10%). At the time of measurement positive end-expiratory pressure was 5 mmHg, the patient was eucapnic and without hypoxia, her mean arterial blood pressure was 80 mmHg, the haematocrit was 34 percent, 30 micrograms norepinephrine per minute was administered and she had received pantoprazole, metamizole, midazolam, fentanyl, propofol, atracurium, metronidazole, cefuroxime, and imipenem.

**Figure 2:**
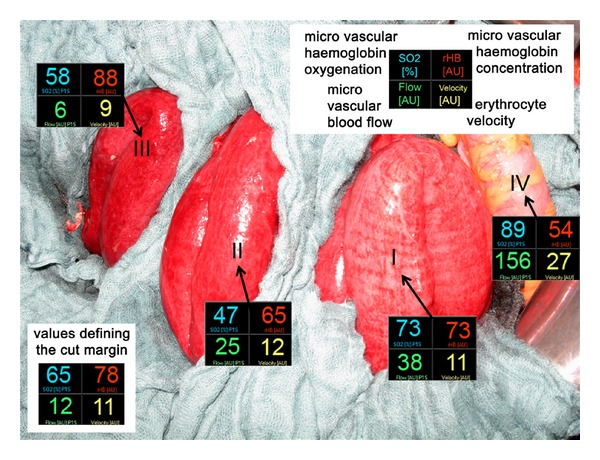
Mean values (10 sec) of laser Doppler flowmetry (microvascular flow and erythrocyte velocity) and spectroscopy (microvascular haemoglobin oxygenation SO_2_ and microvascular haemoglobin concentration rHB) of the proximal segment of the jejunum (I), of the distal segment of the jejunum and the ileum (II and III), and of the transverse colon (IV). The mean values measured at the chosen cut margin are just in range of the critical threshold values. The resected segment of bowel (III) shows a microvascular flow value below the critical threshold value of 10 AU and a microvascular haemoglobin concentration rHB almost at the critical threshold value of 90 AU.

Starting our measurements at the proximal part of the jejunum and proceeding stepwise towards the terminal ileum, we defined the cut margin just before the recommended threshold values were reached as seen in Figure 2. The viable colon was used for reference measurement. Instead of 80 cm we could preserve 190 cm of small bowel. A split stoma was constructed to avoid primary anastomosis.

After operation lactate ion levels decreased to normal values, the stoma remained vital and no further surgical intervention was required. No long-term parenteral nutrition was needed.

**Figure 3:**
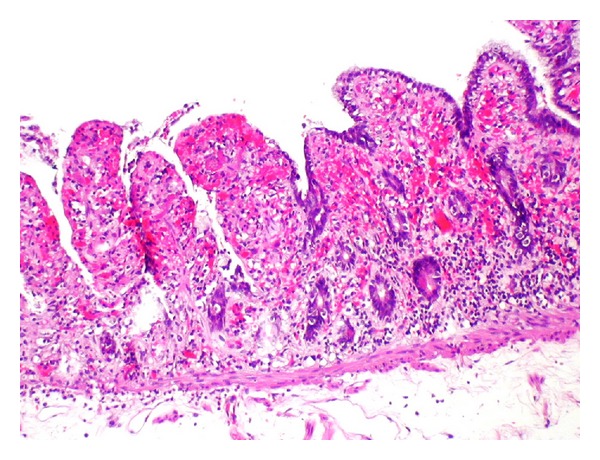
The histologic examination shows a haemorrhagic ischemic necrosis of the mucosa of the smaller intestine with a transmural congestion (corresponding to segment III in the other figures). Only 5 mm of vital mucosa was left next to the cut margin.

The histological examination showed the haemorrhagic ischemic necrosis reaching to the cut margin as near as 5 mm (Figure 3).

## 3. Discussion

As threshold values for this clinical situation are missing, we had to use general threshold values to define the cut margin. Using the viable colon for reference measurement, we could exclude systemic factors having major impact on our measurements.

The measured microvascular haemoglobin value was possibly measured too high due to petechiae and suffusions, but it confirmed our diagnosis of mesenteric venous congestion. The cut margin was finally defined by the values of LDF as the threshold value of microvascular blood flow was reached first.

Using only clinical assessment of bowel viability, we would have left 80 cm of bowel in situ. Thus, we could spare additional 110 cm of small bowel by using LDF and spectrometry. Probably, we were able to avoid short bowel syndrome with all its sequelae. 

We conclude that LDF and spectrometry are potentially powerful methods to assist the surgeon's decision-making in critical venous mesenteric perfusion, thus having an important impact on clinical outcome. However, since the methods are delicate, the measurements have to be done meticulously and the results need to be interpreted carefully. 

## References

[1] Humeau A, Steenbergen W, Nilsson H, Strömberg T (2007). Laser Doppler perfusion monitoring and imaging: novel approaches. *Medical and Biological Engineering and Computing*.

[2] Leung FW (2008). Endoscopic reflectance spectrophotometry and visible light spectroscopy in clinical gastrointestinal studies. *Digestive Diseases and Sciences*.

[3] Redaelli CA, Schilling MK, Büchler MW (1998). Intraoperative laser Doppler flowmetry: a predictor of ischemic injury in acute mesenteric infarction. *Digestive Surgery*.

[4] Oldenburg WA, Lau LL, Rodenberg TJ, Edmonds HJ, Burger CD (2004). Acute mesenteric ischemia: a clinical review. *Archives of Internal Medicine*.

[5] Rhee RY, Gloviczki P (1997). Mesenteric venous thrombosis. *Surgical Clinics of North America*.

[6] Ballard JL, Stone WM, Hallett JW, Pairolero PC, Cherry KJ (1993). A critical analysis of adjuvant techniques used to assess bowel viability in acute mesenteric ischemia. *American Surgeon*.

